# The Effect of Organophosphate Exposure on Neuronal Cell Coenzyme Q_10_ Status

**DOI:** 10.1007/s11064-020-03033-y

**Published:** 2020-04-18

**Authors:** Nadia Turton, Robert A. Heaton, Fahima Ismail, Sioned Roberts, Sian Nelder, Sue Phillips, Iain P. Hargreaves

**Affiliations:** 1 School of Pharmacy and Biomolecular Sciences, Liverpool John Moores University, Liverpool, UK; 2 The Royal Liverpool University Hospital, Royal Liverpool and Broadgreen NHS Trust, Prescot Street, Liverpool, UK

**Keywords:** Organophosphate, Methyl-parathion, Dichlorvos, Chloropyrifos, Mitochondria, Coenzyme Q_10_

## Abstract

Organophosphate (OP) compounds are widely used as pesticides and herbicides and exposure to these compounds has been associated with both chronic and acute forms of neurological dysfunction including cognitive impairment, neurophysiological problems and cerebral ataxia with evidence of mitochondrial impairment being associated with this toxicity. In view of the potential mitochondrial impairment, the present study aimed to investigate the effect of exposure to commonly used OPs, dichlorvos, methyl-parathion (parathion) and chloropyrifos (CPF) on the cellular level of the mitochondrial electron transport chain (ETC) electron carrier, coenzyme Q_10_ (CoQ_10_) in human neuroblastoma SH-SY5Y cells. The effect of a perturbation in CoQ_10_ status was also evaluated on mitochondrial function and cell viability. A significant decreased (P < 0.0001) in neuronal cell viability was observed following treatment with all three OPs (100 µM), with dichlorvos appearing to be the most toxic to cells and causing an 80% loss of viability. OP treatment also resulted in a significant diminution in cellular CoQ_10_ status, with levels of this isoprenoid being decreased by 72% (P < 0.0001), 62% (P < 0.0005) and 43% (P < 0.005) of control levels following treatment with dichlorvos, parathion and CPF (50 µM), respectively. OP exposure was also found to affect the activities of the mitochondrial enzymes, citrate synthase (CS) and mitochondrial electron transport chain (ETC) complex II+III. Dichlorvos and CPF (50 µM) treatment significantly decreased CS activity by 38% (P < 0.0001) and 35% (P < 0.0005), respectively compared to control levels in addition to causing a 54% and 57% (P < 0.0001) reduction in complex II+III activity, respectively. Interestingly, although CoQ_10_ supplementation (5 μM) was able to restore cellular CoQ_10_ status and CS activity to control levels following OP treatment, complex II+III activity was only restored to control levels in neuronal cells exposed to dichlorvos (50 µM). However, post supplementation with CoQ_10_, complex II+III activity significantly increased by 33% (P < 0.0005), 25% (P < 0.005) and 35% (P < 0.0001) in dichlorvos, parathion and CPF (100 µM) treated cells respectively compared to non-CoQ_10_ supplemented cells. In conclusion, the results of this study have indicated evidence of neuronal cell CoQ_10_ deficiency with associated mitochondrial dysfunction following OP exposure. Although CoQ_10_ supplementation was able to ameliorate OP induced deficiencies in CS activity, ETC complex II+III activity appeared partially refractory to this treatment. Accordingly, these results indicate the therapeutic potential of CoQ_10_ supplementation in the treatment of OP poisoning. However, higher doses may be required to engender therapeutic efficacy.

## Introduction

Organophosphate (OP) compounds are used as pesticides and herbicides [1]. They are generally derived from esters of phosphoric acid [2]. These toxic synthetic compounds including dichlorvos, methyl-parathion (parathion) and chloropyrifos (CPF) can cause a neurological syndrome called OP poisoning, which can display itself in both acute and chronic forms [3]. OP poisoning appears to be less prevalent in the UK, compared to developing countries [4]. However, it is becoming increasingly apparent that sheep farmers exposed to OP-based sheep dip are at a high risk of developing OP poisoning [5, 6]. As well as agricultural farmers, air craft cabin crew [7] and military personnel (Gulf war syndrome) are susceptible [8]. In farmers, OP poisoning as a result of exposure to OP based-sheep dip, via both inhalation and direct skin contact, can result in chronic health defects including neurological problems, muscle pain and fatigue [9]. Interestingly similar clinical presentations have been observed in Gulf war syndrome, an illness which developed not long after deployment of war veterans to the 1991 Gulf war [10, 11]. However, although the origin of Gulf war syndrome remains to be uncertain, it has been attributed to exposure of pesticides [12].

The mechanism of action of these OPs involves the inhibition of the nerve transmission enzyme acetylcholinesterase (AChE), which is required for the degradation of acetylcholine [13]. Persistence of acetylcholine at the cholinergic synapses due to AChE inhibition results in insect death due to hyper-excitation [14], and indeed, this also remains the most common adverse side effect of OP exposure in humans [15, 16]. In addition to this, OP poisoning has been associated with mitochondria dysfunction [17]. Clinical presentations have also been observed in OP patients which indicate evidence of mitochondrial dysfunction including fatigue [18], peripheral neuropathy [19] and cerebral ataxia [20]. Interestingly, cerebral ataxia is also a prominent feature of severe Coenzyme Q_10_ (CoQ_10_) deficiency [21, 22].

Support for the potential mitochondrial toxicity of OPs has been provided by identified mitochondrial dysfunction as a result of exposure to OPs. This includes reduction in the activity of mitochondrial electron transport chain (ETC) enzymes, complex I (NADH: ubiquinone reductase), complex II (succinate dehydrogenase: ubiquinone reductase) and complex IV (cytochrome c oxidase) enzymes in rat studies [23], reduction in mitochondrial complex I-III (NADH: cytochrome c reductase) activity, which can in turn affect the production of ATP [24]. Mitochondrial swelling and elevated cytochrome c levels have also been observed in rabbit livers treated with the OP, chloropyrifos [17]. Interestingly, these alterations also appeared to correlate with elevated oxidative stress levels in rabbit’s liver. Furthermore, evidence of oxidative stress as a result of OP exposure has also been observed in vivo as elevations of oxidative stress marker malondialdehyde has been identified in mice blood levels post OP administration [25]. In view of the association between mitochondria dysfunction and cellular oxidative stress [17, 26], the ability of OPs to induce an increase in reactive oxidant species (ROS) generation may further support the potential mitochondrial toxicity of these group of compounds.

The increased oxidative stress and mitochondria dysfunction associated with OP treatment [17], may result from diminution in cellular CoQ_10_ status as the result of exposure to these compounds. CoQ_10_ functions as an electron carrier in the ETC as well as serving as a potent lipid soluble antioxidant [27]. Administration of CoQ_10_ has previously been reported to alleviate mitochondrial oxidative stress [28], as well as enhancing the activities of ETC complexes I-III and complex IV in rats treated with the OP, dichlorvos [24]. CoQ_10_ administration for 12 weeks prior to dichlorvos treatment was also found to improve cognitive performance, mitochondrial physiological features and attenuate neuronal damage [29]. These findings suggest a potential role for CoQ_10_ supplementation in the treatment of OP toxicity. Although, at present there is a paucity of information available on the therapeutic efficacy of CoQ_10_ in the treatment of the clinical symptoms associated with OP poisoning in human subjects. However, veterans suffering with Gulf War syndrome, a condition associated with OP poisoning, have been reported to show some clinical improvement following CoQ_10_ treatment [30]. The factors responsible for biochemical and clinical efficacy of CoQ_10_ in the treatment of OP toxicity have yet to be elucidated. However, the possibility arises that the exogenous CoQ_10_ may be replenishing an endogenous CoQ_10_ deficiency induced by these synthetic compounds.

The present study evaluated the effect of OPs dichlorovos, chloropyrifos and methyl-parathion on human neuronal cellular CoQ_10_ status and mitochondrial function. In addition, the effect of CoQ_10_ supplementation on mitochondrial function following OP exposure was also investigated.

## Materials and Methods

### Materials

All reagents were of analytical grade and obtained from Sigma-Aldrich Chemical (Poole, UK).

### Cell Culture

SH-SY5Y human neuroblastoma cells (passage 17–24) were cultured in Dulbecco's Modified Eagle Medium (DMEM) supplemented with 1% penicillin and streptomycin and 10% foetal bovine serum (FBS). Cultures were maintained in an incubator with 5% CO_2_ at 37 °C. At around 70–90% confluence, cells were harvested, washing twice with phosphate-buffered saline (PBS) followed by the addition of 3 ml of trypsin. Cells were seeded in plastic 96-well plates at a density of 20,000 cells/well or T75 flasks at 150,000 cells/ml prior to treatment with OPs. OP (dichlorvos, chloropyrifos and methyl-parathion) treatment was carried out in 10% DMEM at concentrations of 10 µM, 50 µM and 100 µM and SH-SY5Y cells were treated with the OPs for 5 days. These concentrations were selected in the present study as these OP concentrations have been reported in the blood of individuals who are regularly exposed to OPs [31, 32], as well as in patients who have developed OP poisoning [33]. These concentrations also parallel those used in previous studies [34–36]. Because the OPs were dissolved in ethanol, control cells were treated with the vehicle ethanol. Cells were further supplemented with CoQ_10_ (5 µM) for 2 days with a further addition of the OPs (50 µM and 100 µM) according to the methods by Duberley et al. [37].

### Cell Viability Assay

SH-SY5Y human neuroblastoma cell viability was obtained using the tetrazolium dye MTT (3-(4,5-dimethylthiazol-2-yl)-2,5-diphenyl tetrazolium bromide) assay. This colorimetric assay is based on the ability of the mitochondrial enzyme NADPH-dependant cellular oxidoreductase enzymes to reduce MTT to its purple coloured insoluble formazan. Trypan blue dye solution was added to the cells and dye excluding cells were counted using a haemocytometer. Cells were plated and treated with OPs as previously described. MTT solution (20 µl) was added to each well of the 96 well plate post a 5 day incubation with the OPs. After a 3–4 h incubation with MTT at 37 °C, the formazan product was solubilized using 100 µl of DMSO and the absorbance of each well was read at 570 nm on a CLARIOstar® Plus.

### ETC Enzyme Activities

The activities of the ETC complex II/III (succinate: cytochrome C reductase) and citrate synthase were determined spectrophotometrically at wavelengths of 412 nm and 550 nm respectively, according to methods described by Duberley et al. [38]. Complex II/III activities were expressed as a ratio to citrate synthase activity to account for mitochondrial enrichment [39].

### Total Protein Determination

Cellular protein concentration was determined by the method of Lowry et al. [40]. Protein standards were prepared by diluting BSA (0.2 mg/ml) in H_2_O (0, 25, 50, 75, 100, 200 µg/ml). Samples were diluted 1/100 in H_2_O in Eppendorf tubes. 100 µl of reagent A and 800 µl of reagent B were added to the Eppendorf tubes and standards. Absorbance was measured spectrophotometrically at 750 nm. Protein concentrations can be determined using relative absorbance of protein standards expressed as (mg/ml).

### Lactate Measurement

Following a 5-day incubation with the OPs, 1 ml of culture media from control and OP treated cells was transferred to an Eppendorf tube and frozen at − 80 °C. Samples were thawed and immediately transferred to hospital grade grey topped Fluoride (K EDTA) tubes. The samples were analysed on the Roche Cobas c analyser using Roche 2nd generation LACT2 colorimetric lactate assay. This assay utilises lactate oxidase to oxidise lactate to pyruvate with the generation of hydrogen peroxide. The hydrogen peroxide formed then reacts under a reaction catalysed by a peroxidase with phenol and 4-aminophenazone to form a red-violet quinoneimine dye which absorbs at 500 nm. The resulting increase in absorption is proportional to the amount of lactate present in the sample.

### Quantification of CoQ_10_ Status

The CoQ_10_ status of SH-SY5Y cells was determined using high pressure liquid chromatography with UV detection at 275 nm according to the method of Duncan et al. [41]. The results were expressed pmol/mg of protein.

### Statistical Analysis

All results are expressed as a mean ± standard deviation. One-way ANOVA with Tukey’s multiple comparison post hoc test was used for comparison of groups > 2 using statistical software GraphPad Prism. P < 0.05 was considered significant.

## Results

### Organophosphates Induced a Significant Reduction in Cell Viability

**Fig. 1:**
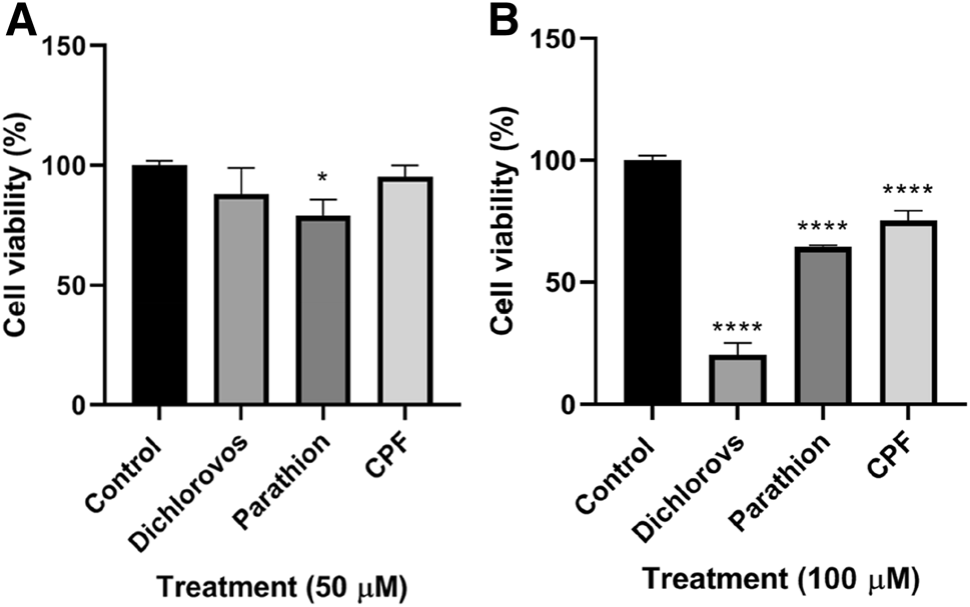
Cell viability (%) of SH-SY5Y cells following a 5 day incubation with OPs dichlorvos, parathion and CPF for concentrations of **a** 50 µM (n = 3) **b** 100 µM (n = 3). Error bars represent standard deviation; statistical analysis was carried out using one-way ANOVA with Tukey’s multiple comparison post hoc test; levels of significance: *p < 0.05, **p < 0.005, ***p < 0.0005, ****p < 0.0001 compared to control levels

The MTT cytotoxicity assay was used to assess cellular metabolic activity post treatment with OPs. The percentage (%) of viable cells following a 5 day incubation with a concentration of 10 µM of the OPs showed very little effect on cell viability (data not shown). However, at 50 µM MTT conversion was reduced significantly by 20% (P < 0.05) for parathion treated cells compared to control levels, which directly corresponds to a significant reduction in cell viability (Fig. 1). Following a 5 day incubation with OPs (100 µM) dichlorvos, parathion and CPF there was an 80%, 55% and 25% (P < 0.0001) significant reduction in cell viability respectively compared to control levels (Fig. 1).

### Organophosphate Treatment had no Effect on Lactate Concentration

**Fig. 2:**
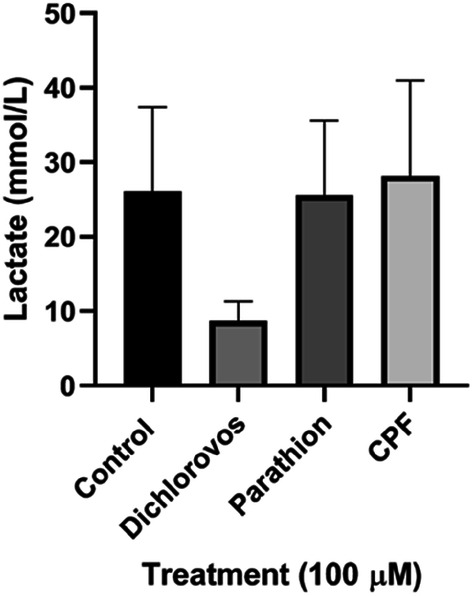
Effect of 5 day treatments of OPs dichlorvos, parathion and CPF (100 µM) on SH-SY5Y cellular medium lactate concentration (mmol/L) (n = 3). Error bars represent standard deviation; statistical analysis was carried out using one-way ANOVA with Tukey’s multiple comparison post hoc test; levels of significance: *p < 0.05, **p < 0.005, ***p < 0.0005, ****p < 0.0001 compared to control levels

Incubation of SH-SY5Y cells with OPs dichlorvos, parathion and CPF (100 µM) resulted in no significant difference in lactate medium concentrations (mmol/l) compared to control levels (Fig. 2).

### Organophosphates Induced Neuronal Coenzyme Q_10_ Deficiency

**Fig. 3:**
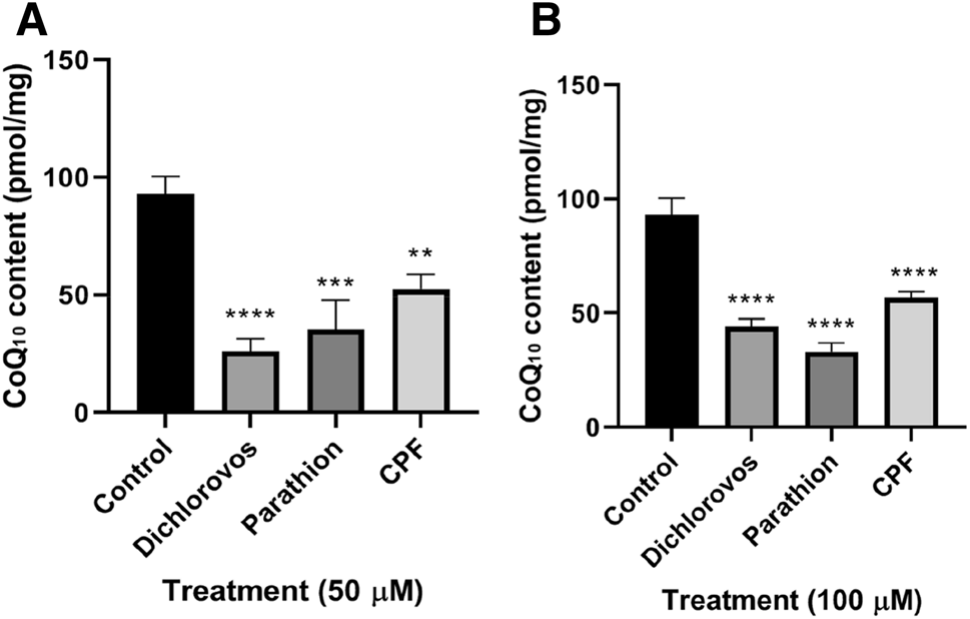
Effect of 5 day treatments of OPs dichlorvos, parathion and CPF on CoQ_10_ content (pmol/mg) in SH-SY5Y cells. **a** 50 µM concentration of OPs (n = 3), **b** 100 µM concentration of OPs (n = 3). Error bars represent standard deviation; statistical analysis was carried out using one-way ANOVA with Tukey’s multiple comparison post hoc test; levels of significance: *p < 0.05, **p < 0.005, ***p < 0.0005, ****p < 0.0001 compared to control levels

The CoQ_10_ status of SH-SY5Y cells decreased following a 5 day incubation with the OPs. Cellular CoQ_10_ status significantly decreased 72% (P < 0.0001), 62% (P < 0.0005) and 43% (P < 0.005) post treatment with dichlorvos, parathion and CPF (50 µM) respectively compared to control levels (Fig. 3). Cellular CoQ_10_ status significantly decreased (P < 0.0001) by 52%, 64% and 40% post treatment with dichlorvos, parathion and CPF (100 µM) respectively compared to control levels (Fig. 3).

### CoQ_10_ Supplementation Demonstrated Protective Properties Against OP-Induced Cellular Death

**Fig. 4:**
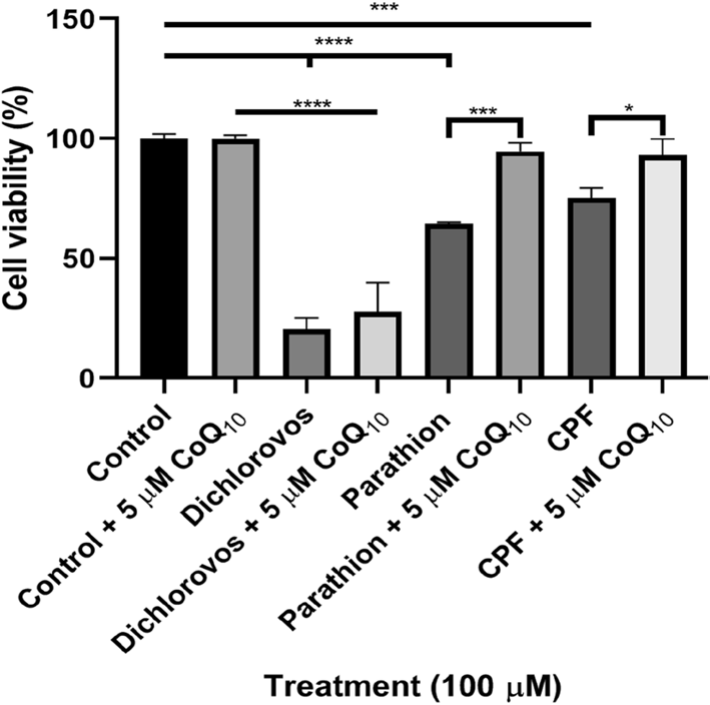
Effect of a 2 day supplementation with CoQ_10_ following a 5 day incubation with OPs dichlorvos, parathion and CPF (100 µM) in SH-SY5Y cells on cell viability (%). Error bars represent standard deviation; statistical analysis was carried out using one-way ANOVA with Tukey’s multiple comparison post hoc test; levels of significance: *p < 0.05, **p < 0.005, ***p < 0.0005, ****p < 0.0001

MTT cytotoxicity assay was used to measure cell viability (%) following 5 day incubations with OPs dichlorvos, parathion and CPF (100 µM), and then a further 2 day incubation with CoQ_10_ (5 µM). Post treatment with CoQ_10_ parathion and CPF treated cells resulted in a significant increase in cell viability, increasing 30% (P < 0.0005) and 18% (P < 0.0001) respectively compared to non-CoQ_10_ supplemented cells (Fig. 4).

### CoQ_10_ Supplementation Improved OP-Induced Alterations on CS Activity

**Fig. 5:**
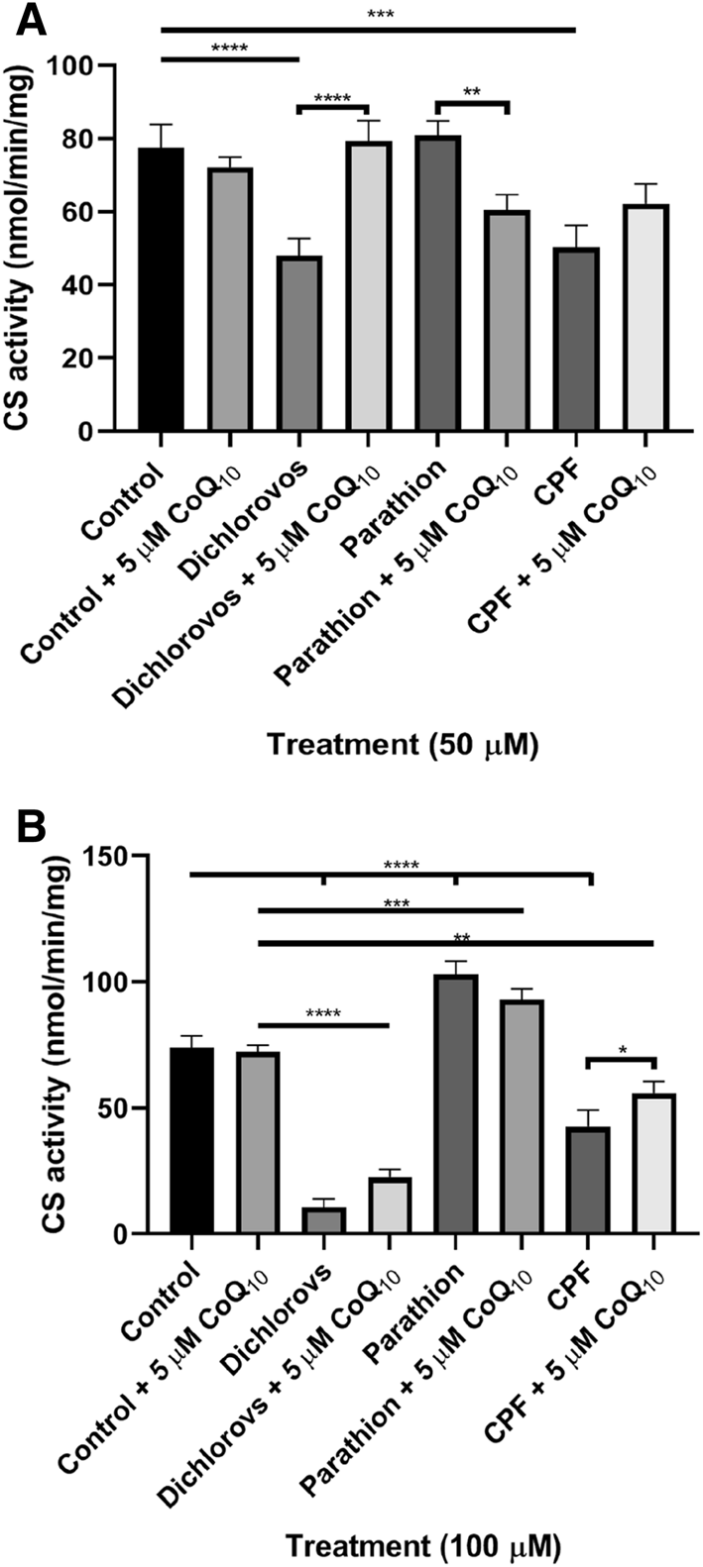
Effect of a 5 day incubation with OPs dichlorvos, parathion and CPF on citrate synthase activity in SH-SY5Y cells, followed by a further 2 day supplementation with CoQ_10_ (5 µM) compared to controls. **a** Citrate synthase activity post treatment with 50 µM of OPs, with and without a further 2 day supplementation with CoQ_10_ (5 µM) (n = 3), **b** citrate synthase activity post treatment with 100 µM of OPs, with and without a further 2 day supplementation with CoQ_10_ (5 µM) (n = 3). Error bars represent standard deviation; statistical analysis was carried out using one-way ANOVA with Tukey’s multiple comparison post hoc test; levels of significance: *p < 0.05, **p < 0.005, ***p < 0.0005, ****p < 0.0001

Incubation of SH-SY5Y cells with OPs dichlorvos, parathion and CPF at 50 µM and 100 µM resulted in alterations in citrate synthase (CS) (mitochondrial matrix marker enzyme) activity. At 50 µM concentrations, dichlorvos and CPF showed a significant reduction in CS activity, decreasing 38% (P < 0.0001) and 35% (P < 0.0005) respectively compared to control levels (Fig. 5). At 100 µM concentrations, dichlorvos and CPF showed a significant reduction (P < 0.0001) in CS activity, reducing 86% and 42% respectively compared to control levels. However, parathion showed a significant increase (P < 0.0001) in CS activity, increasing 39% compared to control levels (Fig. 5).

Post supplementation with CoQ_10,_ CS activity significantly increased by 47% (P < 0.0001) in dichlorvos (50 µM) treated cells compared to non-CoQ_10_ supplemented cells thus showing no significant difference from controls post treatment with CoQ_10_. Post supplementation with CoQ_10_, CS activity significantly increased (P < 0.05) by 20% in CPF (100 µM) treated cells compared to non-CoQ_10_ supplemented cells (Fig. 5).

### CoQ_10_ Supplementation Improved OP-Induced Alterations in Complex II+III Activity

**Fig. 6:**
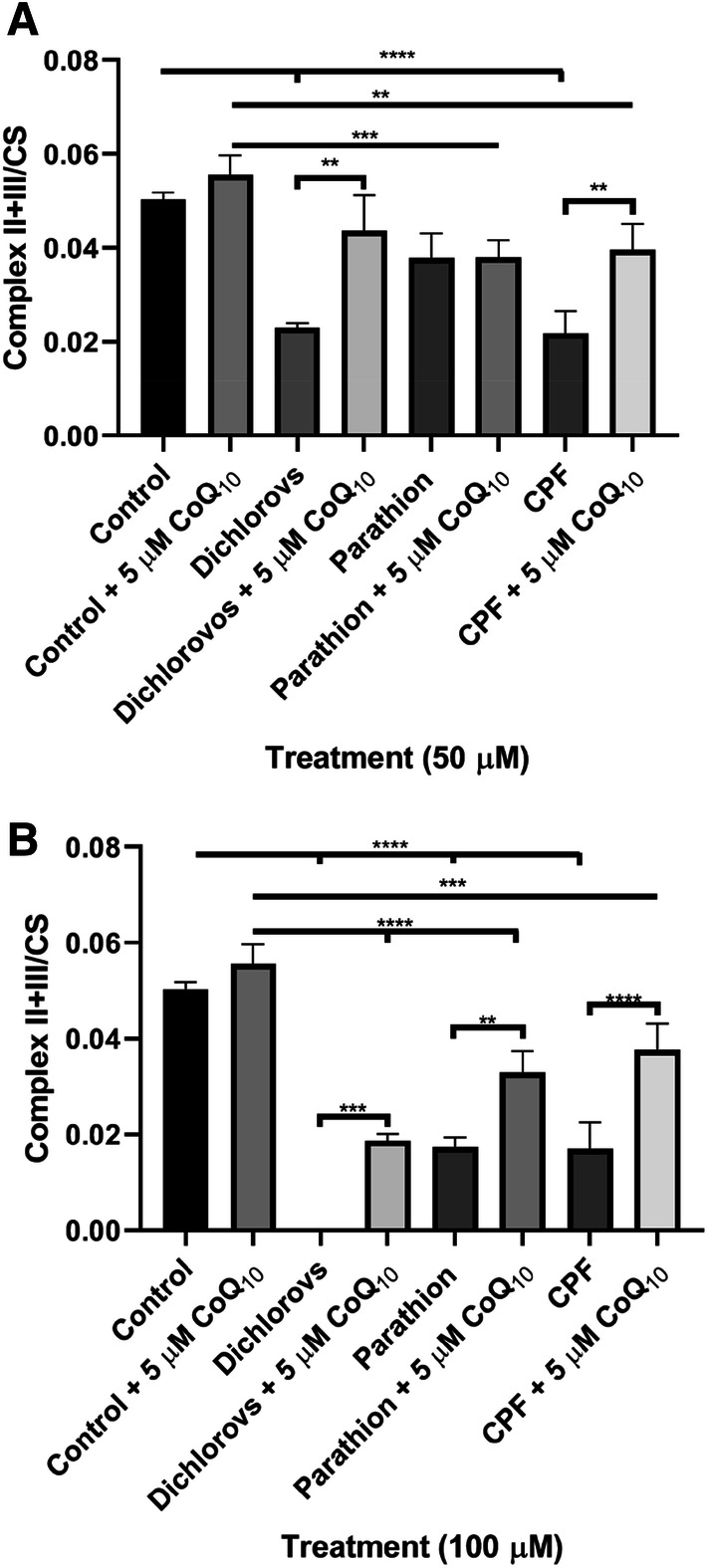
Effect of a 5 day incubation with OPs dichlorvos, parathion and CPF on complex II + III activity in SH-SY5Y cells, followed by a further 2 day supplementation with CoQ_10_ (5 µM) compared to controls. **a** Complex II + III activity post incubation with 50 µM of OPs, with and without a further 2 day supplementation with CoQ_10_ (5 µM) (n = 3), **b** Complex II + III activity post incubation with 100 µM of OPs, with and without a further 2 day supplementation with CoQ_10_ (5 µM) (n = 3). Error bars represent standard deviation; statistical analysis was carried out using one-way ANOVA with Tukey’s multiple comparison post hoc test; levels of significance: *p < 0.05, **p < 0.005, ***p < 0.0005, ****p < 0.0001

Incubation of SH-SY5Y cells with OPs dichlorvos, parathion and CPF at 50 µM and 100 µM resulted in alterations in complex II+III activity. At 50 µM OP concentrations, dichlorvos and CPF showed a significant reduction (P < 0.0001) in complex II+III activity, reducing 54% and 57% respectively compared to control levels. Treatment with 100 µM of dichlorvos showed no complex II+III activity. Parathion and CPF (100 µM) treated cells showed a significant reduction (P < 0.0001) in complex II+III activity, reducing 66% and 67% respectively compared to control levels (Fig. 6).

Post supplementation with CoQ_10_, complex II+III activity significantly increased (P < 0.005), increasing 32% and 28% in dichlorvos and CPF (50 µM) treated cells compared to non-CoQ_10_ supplemented cells. Post supplementation with CoQ_10_, complex II+III activity significantly increased, increasing 33% (P < 0.0005), 25% (P < 0.005) and 35% (P < 0.0001) in dichlorvos, parathion and CPF (100 µM) treated cells compared to non-CoQ_10_ supplemented cells (Fig. 6).

## Discussion

The results of the present study have provided evidence of OP induced neuronal cell CoQ_10_ deficiency which was associated with an impairment of ETC complex II+III activity. As far as the authors are aware, this is the first study to report an association between OP exposure and a deficit in cellular CoQ_10_ status and this may offer an explanation for the ability of CoQ_10_ supplementation to ameliorate some of the neurological symptoms associated with OP poisoning [42]. Furthermore, cerebral ataxia has been associated with OP poisoning which is one of the main clinical presentations of CoQ_10_ deficiency [20].

A deficit in cellular CoQ_10_ status was detected following treatment with OPs dichlorvos, parathion and CPF (50 and 100 µM) indicating the ability of OPs to induce neuronal CoQ_10_ deficiency with dichlorvos, inducing the most pronounced deficit in the status of this isoprenoid. The cause of the cellular CoQ_10_ deficiency associated with OP exposure has yet to be elucidated, and may be due to the ability of these synthetic compounds to directly inhibit the CoQ_10_ biosynthetic pathway or as the result of some other secondary mechanism. OPs associated with increased ROS generation may result in an increase in oxidative catabolism as well as a possible inhibition of enzymes in the CoQ_10_ biosynthetic pathway [17].

In view of the dependence of ETC complex II+III activity upon endogenous CoQ_10_ status [43], we also assessed the effect of OP treatment on complex II+III activity. Interestingly, the decrease in cellular CoQ_10_ status was found to accompany a significant loss in complex II+III activity, supporting the requirement for CoQ_10_ for optimal complex II+III activity. This therefore indicates the potential for a deficit in CoQ_10_ status to impair ETC activity. The activity of complex II+III was expressed as a ratio to CS to account for mitochondria enrichment. Therefore, we can assume that the reduction observed to complex II+III activity reflects impairment to oxidative phosphorylation rather than a loss in mitochondrial number [39]. ETC inhibition generates free radicals, leading to increased oxidative stress [44]. Since the ETC is highly susceptible to oxidative damage and ROS can directly damage mitochondrial enzymes [45], the possibility arises that the loss in complex II+III activity may also have resulted from OP-induced ROS generation [17, 25].

Importantly, the 65% reduction in complex II+III activity observed in chloropyrifos and parathion (100 µM) treated cells may not be sufficient to impair oxidative phosphorylation, as it has been previously reported that complex III activity has to be inhibited by 70–80% before oxidative phosphorylation is compromised [46]. In support of this, the assessed lactate medium levels post treatment with the OPs (100 µM) wasn’t found to be elevated compared to control levels, suggesting an upregulation of glycolytic metabolism was not required to compensate for impairment of oxidative phosphorylation [47]. Alternatively, this may be due to a significant reduction in cell viability due to OP-induced toxicity.

The significant increase in CS activity following treatment of the neuronal cells with parathion (100 µM) would indicate that exposure to this synthetic compound induced an increase in mitochondrial biogenesis in the SH-SY5Y cells, which has been reported to occur as an adaptive response to impaired oxidative phosphorylation [48]. In contrast to the increase in CS activity, the level of neuronal CoQ_10_ was found to decrease following treatment with parathion and since approximately 45% of cellular CoQ_10_ is found within the mitochondria [49], this would suggest that the diminution in cellular CoQ_10_ status does not reflect the loss of mitochondrial enrichment. Contrary to parathion, CPF and dichlorvos induced a loss of neuronal cell CS activity which may reflect a loss of mitochondrial enrichment. A decrease in CS activity has previously been associated with a decrease in cellular ATP production, an increase in ROS production and accelerated cell death [50]. This may explain to some degree the evidence of cellular toxicity and loss of cell viability indicated by the MTT assay following exposure to OPs at concentrations of 50 µM and 100 µM. However, no evidence of toxicity was apparent when OPs were used at a concentration of 10 µM (data not shown).

There is a paucity of information available on the ability of CoQ_10_ to ameliorate some of the adverse clinical effects associated with OP toxicity. However, a study by Bayir et al. [51] reported the ability of CoQ_10_ to attenuate OP induced cardiac toxicity. Furthermore, a recent study by Belousova et al. [42] demonstrated that CoQ_10_ improves neurological function in transient focal brain ischemia in rats [42]. Similarly, the present study showed that CoQ_10_ supplementation (5 µM) was able to restore parathion and CPF (100 µM) treated cell viability to control levels. OPs may induce toxicity to cells by their ability to upregulate ROS production and downregulate antioxidant enzymes [52, 53]. Thus, CoQ_10_ may play roles in ameliorating OP induced toxicity by acting as a free radical scavenger to mitigate DNA damage and lipid peroxidation from OP-induced oxidative stress, as well as elevating activities of antioxidant enzymes including OP detoxifying enzyme, paraxonase [29, 54].

CoQ_10_ supplementation was able to restore CS activity to control levels following treatment with the OPs dichlorvos and CPF (50 µM), but not in cells treated with 100 µM of OPs. Although the precise mechanism by which exogenous CoQ_10_ induces this effect is uncertain, in its ability to act as an antioxidant CoQ_10_ may protect the cell against OP induced oxidative stress and therefore prevent an impairment of oxidative phosphorylation or increased loss of mitochondrial enrichment [55]. However, further work will be required to evaluate the effect of CoQ10 supplementation on OP-induced cellular oxidative stress. Alternatively, CoQ_10_ may activate mitochondria biogenesis as indicated by the study of Jing et al. [56] which reported an increase in the ratio of COX-1 mitochondrial DNA to that of SDH-A nuclear DNA following treatment of cells with CoQ_10_. This would therefore explain the restoration of CS activity (marker for mitochondrial enrichment) following treatment with CoQ_10_ in cells exposed to OPs, dichlorvos and CPF (50 µM).

In addition, CoQ_10_ treatment was found to increase ETC complex II+III activity, however the activity of this enzyme only reached control levels in cells treated with 50 µM of dichlorovos. Treatment with 100 µM of this OP resulted in an apparent complete inhibition of complex II+III activity, possibly reflecting the significant loss of cell viability indicated by the MTT assay. Subsequent CoQ_10_ supplementation was found to increase complex II+III activity to 34% of control levels in neuronal cells treated with 100 µM dichlorovos. The failure of CoQ_10_ supplementation to fully restore neuronal cell ETC complex II+III activity to control levels following exposure to OPs (CPF, parathion and dichlorovos at 100 µM) may indicate that the loss of enzyme activity was not solely the result of a diminution in cellular CoQ_10_ status and possibly the result of some other inhibitory mechanism. However, some studies have suggested that only 11% of an exogenous quinone is able to reach the mitochondrion [57] and therefore the failure of CoQ_10_ supplementation to restore ETC complex II+III activity may be due to the dosage of CoQ_10_ used in the study which was 5 µM. In agreement with this, a study by Duberley et al. [37] reported that a dosage of CoQ_10_ above 10 µM may be required to fully restore ETC enzyme activity following a diminution of CoQ_10_ status. complex II+III was not fully recovered post treatment with OPs (100 µM). However, in view of the MTT results, perbutation of complex II+III may not be sufficient to impair oxidative phosphorylation.

In conclusion, this study for the first time has indicated the possibility that OP exposure may result in a loss of cellular CoQ_10_ status which therefore may be a contributory factor to some of the clinical adverse side effects associated with OP exposure. Supplementation with CoQ_10_ was able to restore OP-induced CS activity and cell viability to control levels in some cases. However, future work to evaluate the effect of OPs on oxidative stress levels and the ability for CoQ_10_ to ameliorate OP-induced oxidative stress is required to fully identify the molecular mechanism by which CoQ_10_ improves mitochondria functioning in the present study. CoQ_10_ supplementation was unable to fully restore ETC complex II+III activity to control levels in this study. This may reflect the dosage of the quinone used and higher doses (> 10 µM) may be required to elicit biochemical efficacy. Thus, targeted strategies aimed at restoring cellular CoQ_10_ status following OP exposure should be considered, although further studies are first required to assess evidence of cellular CoQ_10_ deficiency in patients following OP exposure.

## Abbreviations

OP: Organophosphate
ETC: Electron transport chain
CoQ10: Coenzyme Q10
CS: Citrate synthase
CPF: Chloropyrifos
